# Adjunctive mild controlled hypothermia for moderate necrotizing enterocolitis in very low birth weight infants: a prospective cohort study with external contemporaneous controls

**DOI:** 10.1007/s00431-026-07459-4

**Published:** 2026-10-03

**Authors:** Walusa Assad Gonçalves-Ferri, Vitor Coca Sarri, Cristina Helena F. Ferreira, Maria Eduarda Vidoto Martins, Beatriz Tosetto Nogueira, Jucille Meneses, Felipe Yu Matsushita, Daniela Marques L M Ferreira, Marynea S. do Vale, Paulo de Jesus H. Nader, Fernanda Pegoraro G Melo, Marta David R. de Moura, Marisa Marcia Mussi-Pinhata, Davi Casale Aragon

**Affiliations:** 1 Department of Pediatrics, Ribeirão, Preto Medical School, University of São Paulo, Bandeirantes Avenue 3900, Ribeirão Preto, SP 14049-900 Brazil; 2 Departament of Neonatology, Instituto de Medicina Integral Prof Fernando Figueira, Recife, PE Brazil; 3 Faculty of Medicine, University of São Paulo (FMUSP), São Paulo, SP Brazil; 4 Federal University of Uberlândia, Uberlândia, MG Brazil; 5 University Hospital, Federal University of Maranhão, São Luis, MA Brazil; 6 University Hospital of Canoas, Canoas, RS Brazil; 7 University Hospital, State University of Londrina, Londrina, PR Brazil; 8 School of Health Sciences, Secretariat of State Health of the Federal District, Brasília, DF Brazil; 9 Ribeirão Preto School of Medicine, University of São Paulo, Ribeirão Preto, Brazil

**Keywords:** Necrotizing enterocolitis, Hypothermia, Induced, Infant, Premature, Mortality, Surgical procedures, Operative, Propensity score

## Abstract

Necrotizing enterocolitis (NEC) is a severe condition in preterm infants. Few therapies are available to slow disease progression or reduce the need for surgery. This study aims to assess if mild controlled hypothermia as an adjunctive treatment reduces surgical intervention in preterm infants with NEC. A single-center intervention was conducted from 2019 to 2023 and compared with an external, contemporaneous prospective cohort study. Infants weighing less than 1500 g with moderate NEC were enrolled. One center administered mild hypothermia and served as the intervention cohort. The other seven centers served as contemporaneous controls and provided standard care. The intervention group received mild controlled hypothermia. This targeted a core esophageal temperature of 35.5 °C (± 0.5 °C) for 48 h after NEC diagnosis, in addition to standard medical management. The control group received standard care and maintained normothermia. The primary outcome was the need for surgical intervention due to NEC. All-cause mortality was analyzed using subdistribution hazard ratios (SHR) for competing risks. Inverse probability of treatment weighting (IPTW) adjusted for baseline differences to strengthen causal inference. Among 285 preterm infants, 92 were in the intervention group. This group required fewer surgical interventions (14.13% vs. 46.11%; adjusted relative risk [aRR], 0.30; 95% confidence interval [CI], 0.17–0.53) and showed lower mortality risk (0.59; 95% CI, 0.38–0.93) compared to the control group (*n* = 193). The incidence of severe neurological outcomes was similar between groups; informative missingness was addressed using deterministic MNAR sensitivity analyses. Conditional inference tree analysis identified hypothermia as the primary protective factor against surgery.

*Conclusion*: Mild controlled hypothermia may reduce the need for surgical intervention and improve survival rates, indicating its potential as a promising adjunctive therapy.

**Table:** 

**What is Known**: *• Necrotizing enterocolitis (NEC) affects preterm very low birth weight (VLBW) infants with sufficient severity to require surgical intervention. Mortality rates reach 50% in the most vulnerable subgroups. Current medical management has not demonstrated efficacy in halting disease progression or reducing the need for surgery.* *• Preclinical and pilot studies suggest that adjunctive mild hypothermia (35.5 °C) reduces intestinal inflammation and oxidative stress in NEC. However, no adequately powered multicenter trial has evaluated its clinical effectiveness as an adjunctive therapy.*	
**What is New**: *• Adjunctive mild hypothermia was associated with decreased rates of surgical intervention and reduced all-cause mortality.* *• Exploratory analyses indicated that adjunctive mild hypothermia was the factor most strongly associated with avoidance of surgery*

**Supplementary information:**

The online version contains supplementary material available at https://doi.org/10.1007/s00431-026-07459-4.

## Introduction

NEC is a critical gastrointestinal emergency in preterm infants worldwide, causing high morbidity, mortality, and neurodevelopmental impairment [1–3]. Although advances have been made in understanding pathophysiology and prevention, therapeutic options for established NEC remain limited, necessitating novel interventions [4]. Furthermore, severe NEC often requires surgery, prolonged parenteral nutrition, and high resource utilization, underscoring the need for effective treatments [3–5].

Current management of NEC focuses on acute interventions to limit disease progression and protect infant health [4, 5]. Surgical intervention is necessary in approximately 30–50% of infants and is frequently associated with high mortality and significant complications [5–7]. Investigational therapies, including cell-based and microbiota-targeted treatments, TLR4 inhibitors, and immunotherapy, remain expensive and have been assessed only in preclinical models [8]. Furthermore, remote ischemic conditioning and mild hypothermia have been investigated solely in pilot studies [9–12].

Accidental hypothermia increases morbidity and mortality [13, 14], whereas therapeutic hypothermia has demonstrated efficacy for hypoxic-ischemic encephalopathy (HIE) exclusively in term infants [15]. However, mild therapeutic hypothermia may reduce inflammation and confer cellular protection in inflammatory diseases such as NEC [10, 16–19].

Mild controlled hypothermia involves the deliberate reduction of body temperature to a modest level, such as 35.5 °C, thereby replicating the innate cold-stress response observed during severe physiological stress [16]. This response activates adaptive metabolic mechanisms and may offer protection against NEC, potentially improving survival rates and reducing the need for surgical intervention [10–12, 16–19]. This study evaluated the adjunctive use of mild controlled hypothermia in a large cohort of preterm infants diagnosed with moderate NEC. The objective was to assess whether this intervention reduces the incidence of surgical procedures and all-cause mortality compared to standard neonatal intensive care across multiple level III units.

## Materials and methods

### Study design and setting

A prospective cohort study with external contemporaneous controls was employed. Outcomes were evaluated among infants weighing less than 1500 g with moderate NEC at eight Brazilian level III neonatal intensive care units between 2019 and 2023.

This approach was selected because mild controlled hypothermia had already been incorporated into the standard adjunctive care protocol for moderate NEC at the intervention center, based on previous pilot observations. Conducting a randomized controlled trial across all sites was not feasible due to limited funding for randomization procedures.

A single-center intervention was compared with an external contemporaneous controls approach to evaluate outcomes between the center implementing an established hypothermia protocol and seven control centers providing standard neonatal intensive care without targeted thermal intervention. In situations where randomization is not feasible, concurrent nonrandomized controls represent a practical alternative [20]. To enhance causal inference, inverse probability of treatment weighting (IPTW) was applied to address baseline differences between groups [21].

External controls from multiple centers and rigorously collected prospective data were incorporated to facilitate a robust comparative assessment. Data were obtained from each center’s prospective database within the Vermont Oxford Network (VON). Both control and intervention centers collected and entered data prospectively in accordance with the VON’s stringent guidelines and protocols, which are applicable to all eight level III NICUs [22]. Subsequently, the study data were analyzed retrospectively (Fig SM1: Supplementary material).

**Fig. 1:**
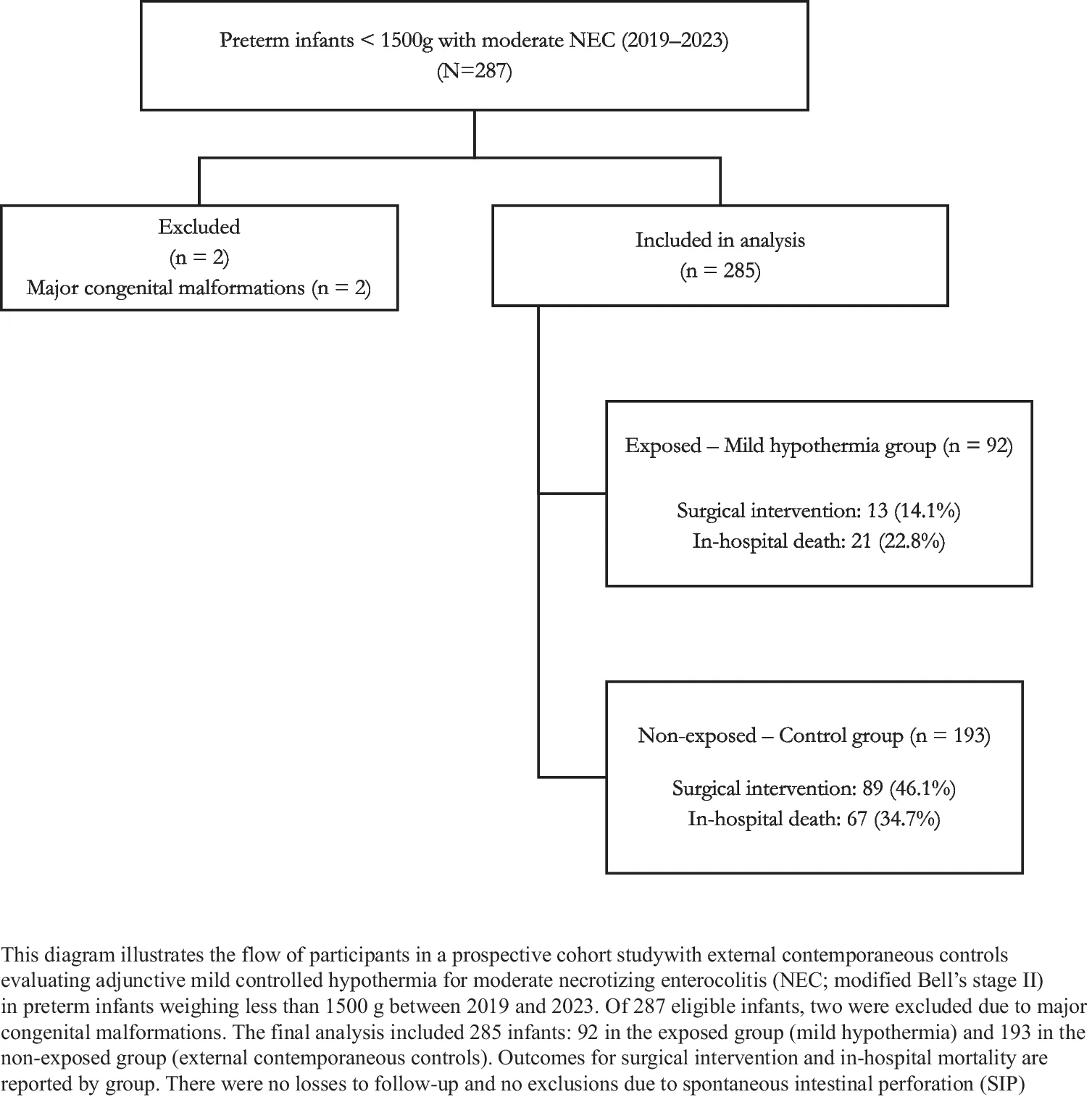
Participant flow diagram

### Participants

Preterm neonates with a birth weight below 1500 g, born and admitted during the study period at participating NICUs, were prospectively monitored by clinical staff.

A formal sample size calculation was not performed because the cohort comprised all eligible infants admitted during the study period via convenience sampling. The study period began with the implementation of the mild controlled hypothermia protocol at the intervention center in 2019 and continued through 2023. This approach optimized the use of available data from the intervention center’s established practice and enabled a comprehensive multicenter comparison within a consistent temporal framework.

The inclusion criteria specified a diagnosis of NEC according to VON criteria, which are similar to the modified Bell’s staging by Walsh [23]. Diagnosis was established by the presence of at least one clinical sign, such as bilious gastric aspirate or emesis, abdominal distension or discoloration, or occult or gross blood in the stool, excluding cases attributable to fissures. Additionally, pneumatosis intestinalis (radiographic finding) was required. To ensure accuracy and clinical validity in data collection and diagnostic processes, all cases underwent independent review and verification by two experienced neonatologists, including one senior neonatologist.

Neonates presenting with major congenital malformations, genetic abnormalities, absence of parental consent, or intraoperatively diagnosed spontaneous intestinal perforation were excluded from the study. To reduce assessment bias, the data analysts were blinded during analysis. Regarding intervention, blinding is not possible due to characteristics of the intervention and study design (single-center study with prospective concurrent controls).

The control group received standard treatment, which included maintaining normothermia with a target body temperature of 36.5–37.5 °C, measured using a skin sensor. Additional interventions comprised gastric decompression via intermittent nasogastric suction, parenteral nutrition, and fasting from enteral feeds for at least 7 days after diagnosis or until both clinical and radiological resolution were achieved. Broad-spectrum antibiotic therapy was administered according to institutional protocols, typically consisting of vancomycin and gentamicin, with metronidazole added for anaerobic coverage. The intervention group received the same standard treatment and, in addition, underwent mild hypothermia, with temperature measured using an esophageal probe.

### Mild controlled hypothermia protocol

The mild hypothermia protocol cooled patients to 35.5 °C (± 0.5 °C) for 48 h. All incubator heating sources were deactivated, and the room temperature was maintained at 24 °C (± 1 °C) using automated controls. Patient temperature was monitored with a transesophageal probe connected to a multi-parameter monitor (Dixtal Dx 2022). Temperature and vital signs were recorded every 15 min. If the patient’s temperature fell below the target, the incubator’s heating mechanism was activated. Additional cooling was achieved by placing ice packs under the mattress when necessary. A designated research nurse ensured adherence to the protocol, documenting all measurements, interventions, and deviations on standardized case report forms.

Cooling was applied at 0.5 °C per hour until the target temperature was reached. The attending nurse regulated the temperature reduction by measuring the temperature every 15 min during both the cooling and rewarming phases and by adjusting the incubator’s servo-control set-point in a stepwise manner to maintain a consistent cooling rate of approximately 0.5 °C per hour until the target esophageal temperature of 35.5 °C (± 0.5 °C) was attained. Rewarming was performed at a controlled rate of 0.5 °C per hour by incrementally increasing the incubator’s servo-control set-point.

Cardiac rhythm and vital signs were continuously monitored. Rewarming was performed at 0.5 °C/h until 36.5 °C was reached, utilizing the incubator’s servo-control system. The Neonatal Infant Pain Scale (NIPS) was applied at the time of NEC diagnosis. If the NIPS score exceeded 3, fentanyl was administered at 0.5 mcg/kg/h for analgesia. During hypothermia, the COMFORT scale was used to assess analgesia adequacy, and fentanyl dosing was adjusted accordingly. Adequate analgesia was defined as a COMFORT score of 17–26.

The intervention center demonstrates experience and technical proficiency in maintaining precise thermal control during mild hypothermia, as validated in published safety and feasibility studies [11, 12]. In these studies, the low-technology cooling technique achieved high stability, with esophageal temperatures consistently maintained within the target range of 35.5 °C ± 0.5 °C over the 48-h intervention period. This expertise ensured that the thermal management protocol in the current study was implemented with comparable rigor, maintaining patients within the intended hypothermic range.

### Variables and definitions

Patient characterization included documentation of antenatal corticosteroid administration (systemic corticosteroids administered before birth at any dose or drug, including both complete and incomplete cycles), maternal hypertension, early-onset sepsis (defined as recovery of a bacterial pathogen from blood or cerebrospinal fluid cultures obtained within the first three days of life), presence of maternal chorioamnionitis as assessed by obstetric staff, sex, gestational age determined by first-trimester ultrasound or New Ballard score, birth weight at delivery, and Apgar scores at 1 and 5 min.

### Outcomes

The primary outcomes assessed were the need for surgical intervention and mortality. The surgical management protocol for NEC was standardized and consistently implemented across all participating level III neonatal intensive care units (NICUs). The criteria for surgical intervention were explicitly defined: (1) pneumoperitoneum, indicated by radiographic evidence of bowel perforation, (2) positive paracentesis, strictly defined as the aspiration of stool or bile.

NEC was diagnosed when abdominal radiographs demonstrated pneumatosis intestinalis, portal venous gas, or fixed bowel loops, typically accompanied by systemic inflammatory signs and metabolic acidosis.

Differentiation between NEC and spontaneous intestinal perforation (SIP) was achieved using defined clinical and radiological criteria. SIP was identified in cases presenting with isolated pneumoperitoneum, often characterized by a gasless abdomen and absence of prior radiological evidence of NEC or significant systemic illness.

For infants who did not undergo surgical intervention, diagnosis relied on clinical progression and serial abdominal imaging. To minimize misclassification, cases that initially presented with pneumoperitoneum but lacked characteristic inflammatory markers of NEC and exhibited clinical stability within 48 to 72 h were classified as SIP. Conversely, cases with systemic instability or multi-organ involvement were categorized as NEC.

### Assessment of data quality and variable harmonization

The data utilized in this study are considered highly reliable. All variables were collected prospectively in the Vermont Oxford Network (VON) database, using standardized definitions and data-entry protocols across all participating centers. Before outcome analyses, two investigators conducted a systematic data quality audit to ensure comparability across centers. During this audit, each variable definition was verified with the coordinators of every participating center to confirm consistency in diagnostic criteria and variable definitions. If a center did not apply a variable definition identical to those of other centers, the corresponding variable was excluded from the specific analysis, while the center itself remained in the study for other variable assessments. Centers were excluded only if variables related to study outcomes, such as death or NEC surgery, did not align with the predefined diagnostic classification. This approach ensured that analyses were based on uniformly defined and validated data, thereby enhancing the reliability and internal validity of the study findings.

### Bias

To reduce assessment bias, data analysts, including statisticians and coders, were blinded to group allocation during analysis. Due to the inherent limitations of blinding in a nonrandomized intervention, additional strategies were employed to minimize performance bias and enhance the reliability and validity of outcome data. To further address potential bias in surgical indication, all surgical cases underwent retrospective review by the center’s neonatologists to confirm the appropriateness of the surgical indication.

Mortality was defined as all-cause death occurring at any point during hospitalization. All participating units provided continuous pediatric surgical services.

Secondary outcomes included cystic leukomalacia and grade III or IV intraventricular hemorrhage, both documented by cranial ultrasound according to the Volpe criteria [24]. Routine cranial ultrasounds were performed on days 7 and 28 of life, with additional imaging as clinically indicated.

### Statistical analysis

A comprehensive description of study variables was provided. Crude (RR) and adjusted (aRR) relative risks between groups were estimated using Poisson regression models with robust variance. These estimates were initially unadjusted. Adjustments were then made using inverse probability of treatment weighting (IPTW). Propensity scores based on gestational age, birth weight, sex, hypertension, chorioamnionitis, antenatal steroid use, 5-min Apgar score were used to derive the weights. This approach aimed to reduce confounding bias in observational data [25].

Primary analyses of neurological outcomes, specifically severe intraventricular hemorrhage (IVH) grades 3–4 and periventricular leukomalacia (PVL), were performed using a complete-case approach. Due to differential missing data in the control group (16% compared to 0% in the intervention group), which resulted from early death before protocolized ultrasound or discharge without imaging, deterministic sensitivity analyses were conducted to evaluate potential informative missingness (missing not at random, MNAR). In the cause-specific scenario (M1), patients who died before imaging were classified as having the outcome, while those discharged without imaging were considered outcome-free. In the worst-case scenario (M2), all missing cases were classified as having the outcome, whereas in the best-case scenario (M3), all missing controls were imputed as not having the outcome. Across all scenarios, no significant differences in neurological outcomes were observed between the groups (Table SM2: Supplementary Material).

Covariate balance after applying propensity score weights was assessed using standardized mean difference (love) plots. These plots enable visual verification of group comparability regarding confounding variables. Prior to weighting, some covariates showed standardized differences exceeding 0.1. This indicated an imbalance. After IPTW, all standardized differences were reduced to near zero and stayed below the 0.1 threshold. This demonstrated excellent balance. The maximum absolute standardized difference after weighting was 0.04, quantifying the improvement in covariate balance. The love plot thus provided graphical and quantitative evidence that weighting effectively minimized confounding biases. This reduced confounding bias from measured baseline covariates of the relative risk estimates for the primary outcome (surgery for NEC).

Hospital mortality and length of hospital stay were evaluated using a competing risk framework. This approach recognizes that hospital death could preclude discharge. Cumulative incidence functions were estimated using Gray’s method (1988). Group effects were quantified by the subdistribution hazard ratio (SHR), according to the Fine & Gray model [26]. The SHR represents the ratio of event rates (death) between groups, adjusted for competing events such as hospital discharge. An SHR greater than 1 indicates a higher risk of death before discharge in the control group. For example, an SHR of 1.5 indicates a 50% higher estimated risk of death before discharge in the control group compared to the hypothermia group. This accounts for the possibility of being discharged alive. The SHR was adjusted using IPTW.

Conditional inference trees were constructed in an exploratory manner to identify potential predictors and interactions associated with surgical intervention. These trees perform recursive partitioning based on conditional statistical tests, which reduces variable selection bias and overfitting. However, because the top split was group assignment (center), the results are descriptive and hypothesis-generating rather than causal. All statistical analyses were conducted using R software version 4.5.0.

#### Ethics

The study was approved by the Institutional Review Board of Ribeirão Preto Medical School, University of São Paulo (FMRP-USP, CAAE number 29879620.5.0000.5440, approval number 3.920.223) and conducted in accordance with the STROBE guidelines [27]. All patients underwent mild hypothermia following signed parental consent.

## Results

A total of 285 preterm infants diagnosed with moderate NEC were enrolled, with 92 infants assigned to the intervention group (mild hypothermia) and 193 to the control group (standard treatment). Two patients were not enrolled due to major malformations (Fig. 1). No cases were excluded from the study based on a diagnosis of SIP.

### Baseline demographic and clinical characteristics

Table 1 summarizes the baseline demographic and clinical characteristics of study participants and indicates comparable profiles across groups. The mean gestational age was 28.4 weeks (SD 2.6) in the hypothermia group and 28.8 weeks (SD 2.8) in the control group (*p* = 0.37). Birth weights were also similar, with means of 957.99 g (SD 269.3) and 1013.76 g (SD 251.8), respectively (*p* = 0.10).

**Table 1:** Baseline demographic and clinical characteristics of study participants by group

Characteristic	Hypothermia group (*n* = 92)	Control group (*n* = 193)	*p* value^a,b^
**Categorical variables** (**no. (%))**			
Male sex	43/92 (46.7%)	99/193 (51.3%)	0.30^a^
SGA	29/89 (33.6%)	67/187 (35.8%)	0.87^a^
Maternal hypertension	42/92 (45.6%)	84/192 (43.7%)	0.80^a^
Chorioamnionitis	16/91 (17.5%)	27/193 (13.9%)	0.47^a^
Antenatal steroid use	79/91 (86.8%)	150/190 (78.9%)	0.14^a^
Early-onset sepsis	8/92 (8.7%)	5/139 (3.6%)	0.09^a^
Cesarean	63/92 (68.4%)	137/193(70.9%)	0.67^a^
**Continuous variables (mean (SD); median (Q1, Q3))** *n* = 92/*n* = 193			
Gestational age (weeks)	28.4 (2.6) 28.8 (26.4, 30.0)	28.8 (2.8) 28.8 (26.7, 30.5)	0.37^b^
Birth weight (grams)	957.9 (269.3) 922.5 (752.5, 1195.0)	1013.7 (251.8) 1000.0 (845.0, 1235.0)	0.10^b^
SNAPPE-II	29.8 (17.9) 29.0 (17.0, 41.0)	26.7 (20.9) 25.0 (10.0, 39.0)	0.09^b^
5-min Apgar score	7.9 (1.8) 8.0 (7.0, 9.0)	7.7 (1.6) 8.0 (7.0, 9.0)	0.16^b^

*SD* standard deviation, *Q1* first quartile (25th percentile), *Q3* third quartile (75th percentile). ^a^*p* values for categorical variables used Fisher’s exact test. ^b^*p* values for continuous variables used the Wilcoxon rank-sum test

After inverse probability of treatment weighting (IPTW) adjustment, all standardized differences were reduced to near zero and remained below the 0.1 threshold, indicating adequate covariate balance. The love plot visually confirms this balance and demonstrates that weighting minimizes confounding bias. This adjustment enhances the causal validity of the relative risk estimates for the primary outcome, surgery for NEC (see Supplementary material—Figure SM2).

Although some unadjusted *p*-values in Table 1 indicated minor numerical differences, IPTW effectively balanced all relevant covariates, including gestational age, birth weight, sex, antenatal steroid use, and 5-min Apgar score. After weighting, all standardized mean differences remained below 0.1 (love plot), demonstrating robust covariate balance and reduced confounding bias in the adjusted risk ratio estimates.

Baseline demographic and clinical characteristics of study participants by center are provided in the Supplementary Material (Table SM-1).

#### Assessment of control group homogeneity

The homogeneity of the external contemporaneous control group was assessed by comparing baseline characteristics across seven control centers (Table SM-1). Most variables, including male sex (*p* = 0.92), maternal hypertension (*p* = 0.31), chorioamnionitis (*p* = 0.07), early-onset sepsis (*p* = 0.15), small for gestational age (SGA, *p* = 0.38), gestational age (*p* = 0.25), birth weight (*p* = 0.23), and 5-min Apgar score (*p* = 0.19), were distributed similarly across centers.

However, antenatal steroid use (*p* < 0.01), mode of delivery (*p* < 0.01), and SNAPPE-II score (*p* < 0.01) demonstrated significant differences. Center C2 was distinct from the other centers regarding the proportion of cesarean deliveries (43.7% compared to approximately 70%). For SNAPPE-II scores, only center 2 had a higher mean score (38.5). SNAPPE-II score greater than 39 was used as an independent variable in the mortality analysis.

Antenatal steroid use and selected SNAPPE-II variables (birth weight, Apgar score at 5 min, gestational age) were incorporated into the propensity score model to calculate the inverse probability of treatment weights (IPTW). Following weighting, all standardized mean differences between the intervention and control groups were reduced to below 0.1 (maximum absolute SMD = 0.04; Figure SM-2).

#### Clinical outcomes

The intervention group demonstrated a significant reduction in NEC-related surgical interventions, with 14.13% of infants requiring surgery compared to 46.1% in the control group. The crude and adjusted risk ratios were 0.31 (95% CI 0.16 to 0.53) and 0.30 (95% CI 0.17 to 0.53), respectively (Table 2). All surgical cases were indicated based on the presence of pneumoperitoneum or positive paracentesis.

**Table 2:** Clinical outcomes in the intervention and control groups

Outcome	Group		Crude risk ratio (95% CI)	Adjusted risk ratio (95% CI)
	Hypothermia *n *(%)	Control *n *(%)		
Surgery	13/92 (14.13%)	89/193 (46.11%)	0.31 (0.16; 0.53)	0.30 (0.17; 0.53)
Mortality	21/92 (22.83%)	67/193 (34.72%)	0.65 (0.39; 1.05)	0.59 (0.38; 0.93)
Severe intraventricular hemorrhage (IVH)^c^	10/92 (10.87%)	9/162 (5.56%)	1.95 (0.79; 4.93)	2.16 (0.88; 5.27)
Periventricular leukomalacia (PVL)^d^	14/92 (15.22%)	16/160 (10.00%)	1.52 (0.73; 3.12)	1.72 (0.86; 3.43)

*CI* confidence interval, *SD* standard deviation, *Q1* first quartile (25th percentile), *Q3* third quartile (75th percentile). ^a^Adjusted risk ratio was calculated using inverse probability of treatment weighting (IPTW). ^b^Necrotizing enterocolitis. ^c^Severe intraventricular hemorrhage (≥ grade III). ^d^Periventricular leukomalacia (grade III)

During hospitalization, all-cause mortality was 22.83% in the intervention group and 34.72% in the control group, indicating a reduction in-hospital mortality (aRR 0.59 (95% CI 0.38 to 0.93)).

#### Length of hospital stay (LOS)

**Fig. 2:**
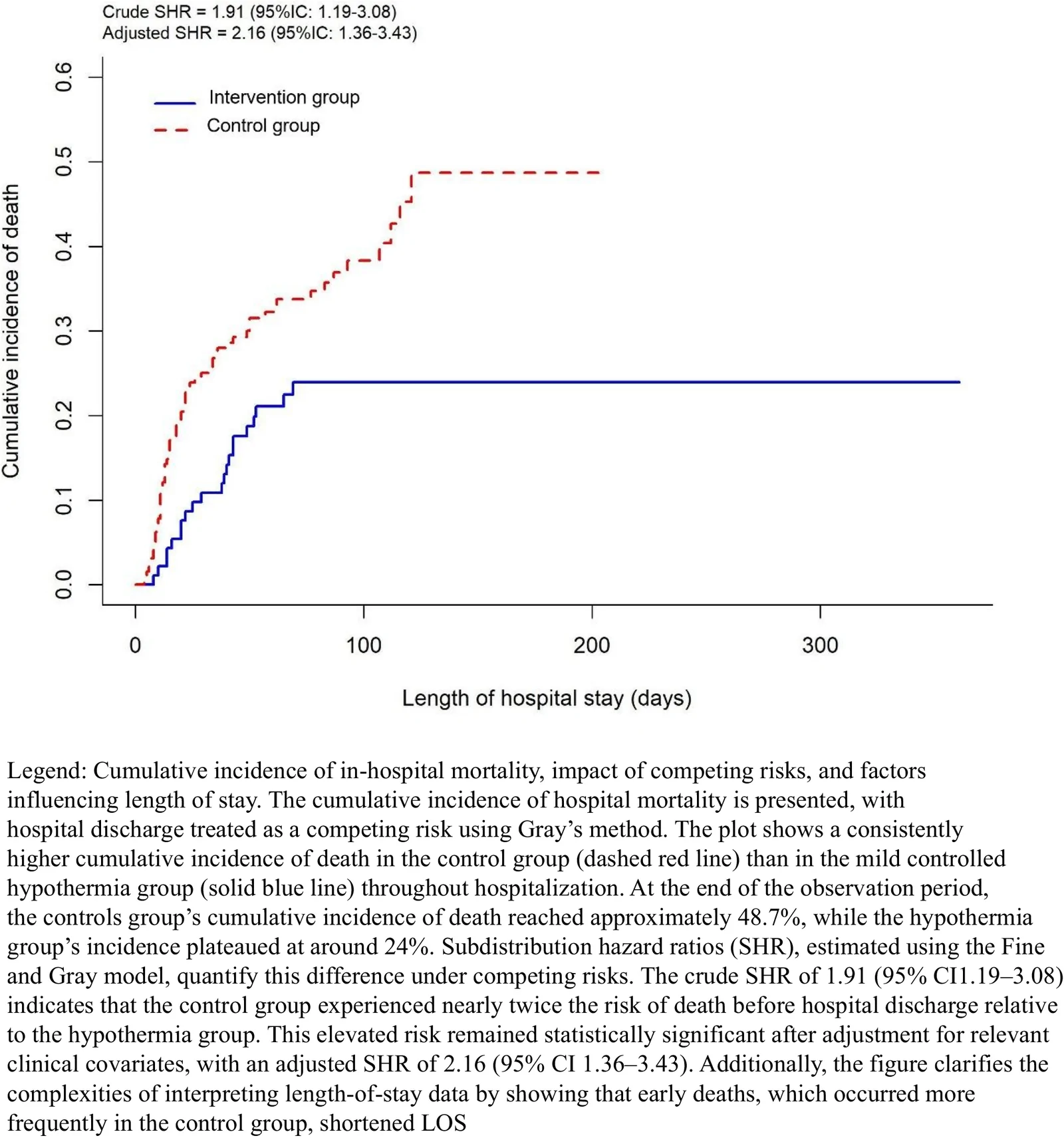
Cumulative incidence of in-hospital mortality and length of stay among preterm infants with necrotizing enterocolitis: competing risks analysis of mild controlled hypothermia using subdistribution hazard ratios

The overall LOS, including both survivors and non-survivors, was longer in the intervention group (mean 81.79 days [SD 51.97], median 73.50 days [Q1, Q3 52.00, 101.50]) than in the control group (mean 60.18 days [SD 45.73], median 49.0 days [Q1, Q3 18.0, 92.0]; *p* = 0.01). This difference is due to lower in-hospital mortality in the hypothermia group, leading to more patients surviving and longer stays. Among survivors (71 in the intervention group and 126 in the control group), the mean hospital stay was also significantly longer in the hypothermia group (95.9 days [SD 50.2]) compared to the control group (76.1 days [SD 44.7]; *p* = 0.01). (Fig. 2).

The LOS is a non-fatal event that can act as a competing risk, potentially precluding observation of in-hospital death. To address this, the risk of death was estimated using Gray’s method and subdistribution hazard ratios (SHR), which account for competing risks. A crude SHR of 1.91 (95% CI 1.19–3.08) indicates that the control group had nearly double the risk of dying before discharge compared to the hypothermia group, suggesting the intervention reduced in-hospital mortality. This result was confirmed by an adjusted SHR of 2.16 (95% CI 1.36–3.43). By the end of observation, the estimated cumulative incidence of death was approximately 48–49% for controls and plateaued at 24–25% for the hypothermia group (Fig. 2), further supporting that mild hypothermia improved survival outcomes.

#### Neurological outcomes

No significant differences in the incidence of severe intraventricular hemorrhage or periventricular leukomalacia were observed between the groups. The control group, however, had substantial missing data for these outcomes (16.06% and 17.10%, respectively), primarily due to early mortality before neuroimaging. To address potential survivorship bias, deterministic sensitivity analyses with cause-specific imputation were conducted across three scenarios (Supplementary Material, Table SM2). In the clinically plausible scenario (M1), controls who died before neuroimaging were imputed as having the outcome, while those discharged without imaging were imputed as outcome-free; the adjusted risk ratios were close to unity (aRR 1.03, 95% CI 0.46–2.29 for severe IVH; aRR 1.05, 95% CI 0.55–2.01 for PVL). In the worst-case scenario (M2), where all missing controls were imputed as having the outcome, the estimates were below unity (aRR 0.69, 95% CI 0.33–1.43 for IVH; aRR 0.80, 95% CI 0.43–1.49 for PVL). In the best-case scenario (M3), where all missing controls were imputed as outcome-free, representing the most favorable scenario for the observed association, the results remained statistically non-significant (aRR 1.70, 95% CI 0.66–4.38 for IVH; aRR 1.84, 95% CI 0.86–3.89 for PVL). (Supplementary Material, Table SM2).

#### Conditional inference tree

**Fig. 3:**
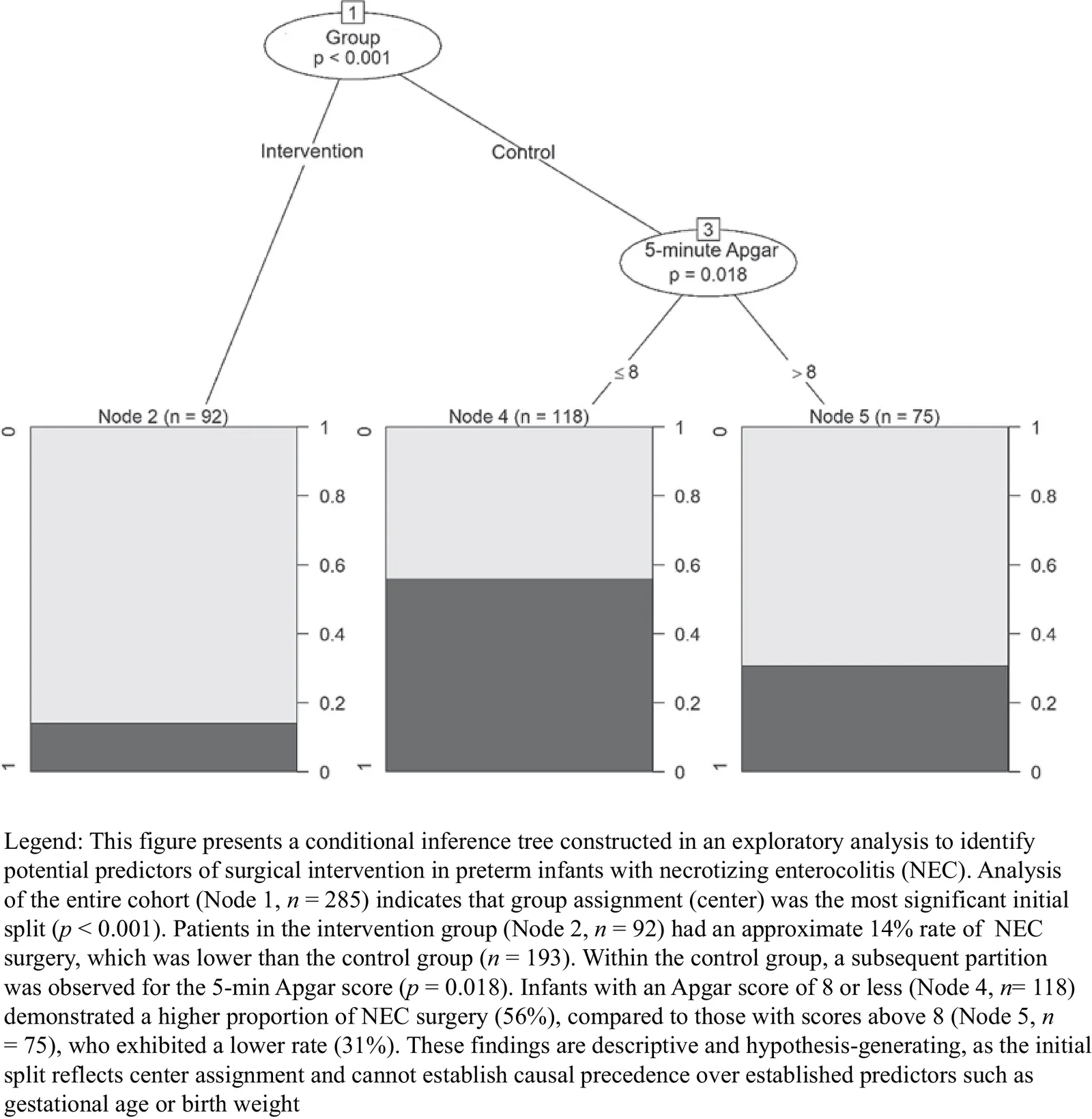
Conditional inference tree for predicting surgical intervention in preterm infants with necrotizing enterocolitis

A conditional inference tree was used in an exploratory analysis to identify potential predictors of NEC surgery (Fig. 3). Group assignment (center) emerged as the primary partition (*p* < 0.001). Assignment to the intervention group (branch ‘1’) was associated with the lowest observed NEC surgery rate, approximately 15%. The control group (branch ‘2’) was further stratified by Apgar score at 5 min (*p* = 0.018). Within this group, an Apgar score of 8 or less (Node 4, *n* = 118) was associated with a higher NEC surgery rate of 55–60%. In contrast, participants with scores above 8 (Node 5, *n* = 75) exhibited a lower rate, approximately 30% (Fig. 3). These findings are descriptive and hypothesis-generating, as the initial split reflects center assignment and cannot establish causal precedence over established predictors such as gestational age or birth weight.

## Discussion

Mild controlled hypothermia in preterm infants with moderate NEC reduced surgical interventions by threefold and significantly lowered hospital mortality compared to standard care. This large, diverse multicenter cohort supports this novel treatment approach and confirms previous single-center findings [10, 11]. Reducing surgical NEC is essential, as its complications increase healthcare costs and negatively impact long-term quality of life [2, 3].

The incidence of NEC varies across centers due to multiple contributing factors. The intervention center has consistently reported higher NEC rates, primarily because it functions as a specialized quaternary referral center for high-risk and pathological pregnancies, including cases of severe maternal hypertension and multiple gestations. This results in a baseline population of neonates with increased biological vulnerability. In addition, nutritional and constitutional factors likely influence the incidence of NEC. The intervention center serves a population characterized by a high prevalence of Small for Gestational Age (SGA) infants and documented nutritional deficiencies. Furthermore, studies conducted at this unit have reported lower levels of docosahexaenoic acid (DHA) in breast milk compared to international standards, a factor known to affect intestinal integrity and the inflammatory response in preterm infants [28, 29].

The rationale for mild hypothermia treatment is based on the adaptive transition between hyperthermia and hypothermia. This evolutionary process balances the pathogen-clearing effects of fever with the energy-conserving, tissue-protective benefits of hypothermia during infection [30]. Fever enhances leukocyte movement and innate immune responses, while hypothermia serves as a disease-tolerance mechanism in severe sepsis or systemic inflammatory response syndrome (SIRS). In these situations, hypothermia conserves energy, induces metabolic quiescence, reduces oxygen consumption, and protects organs from hypoxia and neutrophil-mediated damage when fever cannot be maintained [31]. Therapeutic hypothermia near 35.5 °C provides optimal protection by modulating pro-inflammatory responses and preserving immune surveillance. This adaptive transition is a coordinated strategy to resolve infection and preserve tissue, rather than a sign of physiological failure [30, 31].

Furthermore, studies in neonatal models demonstrate that maintaining a temperature of 33.5–34.5 °C reduces oxidative stress by lowering malondialdehyde levels and sustaining glutathione (GSH) homeostasis in the intestinal mucosa [32, 33]. Hypothermia thereby protects the neonatal gut during the acute phase [34, 35]. Clinical studies further indicate that systemic cooling is safe for neonates with advanced NEC and may modulate inflammation [10, 11].

The present findings align with our previous within-center before-and-after evaluation from the NEOTHERM initiative [10, 11], in which preterm infants with moderate-to-severe NEC (modified Bell’s stage II/III) treated before (2015–2018) and after (2018–2020) implementation of low-technology mild controlled hypothermia (35.5 °C for 48 h) showed reduced surgery (78.9 to 25.0%; aRR, 0.40; 95% CI, 0.19–0.85), intestinal perforation (73.7 to 12.5%; aRR, 0.39; 95% CI, 0.18–0.83), extensive intestinal resection (0% vs. 15.7%), NEC-related mortality (0% vs. 31.6%; *p* < 0.01), and shorter parenteral nutrition duration (aHR, 5.28; 95% CI, 1.88–14.89) [11]. A subsequent safety analysis confirmed that this cooling technique was feasible and not associated with dysthermia, hemodynamic instability, bleeding, seizures, or adverse laboratory or ventilatory changes [12].

Previous studies have demonstrated high mortality and surgical intervention rates in NEC. Multicenter studies indicate that 27 to 52% of preterm infants diagnosed with NEC require surgery [36]. A systematic review reported mortality rates of 23.5% for confirmed NEC, 34.5% for surgical NEC, 30.1% for infants under 1500 g with Bell stage IIA, 40.5% for those under 1000 g, and 50.9% for extremely low birth weight (ELBW) surgical cases.^37^ In the present study, all-cause mortality was evaluated due to the characteristics of the available data. The study population comprised infants under 1500 g, a group for whom higher mortality rates have been documented in the literature [37, 38]. Despite this, the control group exhibited a 34.7% all-cause mortality rate, whereas the intervention group exhibited a 22.7% all-cause mortality rate.

Therapeutic hypothermia in preterm infants raises concerns about potential neurological effects due to the susceptibility of the developing brain to thermal and hemodynamic disturbances. The present study identified no significant differences in the incidence of severe intraventricular hemorrhage or periventricular leukomalacia between the intervention and control groups, a result consistent across all analytical scenarios. In the clinically plausible imputation scenario (M1), adjusted risk ratios approximated unity. In the worst-case scenario (M2), where all missing controls were assumed to have the outcome, the estimates were below unity. In the best-case scenario (M3), where all missing controls were assumed to be outcome-free, the estimates remained statistically non-significant. None of the scenarios demonstrated adverse neurological outcomes attributable to hypothermia. However, due to the observational design of this study, these findings should be interpreted with caution. Larger, adequately powered randomized controlled trials are necessary to provide conclusive evidence.

In exploratory conditional inference tree analysis, group assignment (center) appeared as the primary partition associated with surgical rates. Within the control group, a low 5-min Apgar score was identified as a secondary factor associated with increased surgical intervention. This observation is consistent with previous studies indicating that 5-min Apgar scores below 8 are correlated with a higher incidence and severity of NEC due to poor cardiorespiratory adaptation and diminished physiological reserve. However, because the initial split reflects center assignment, these findings are descriptive and hypothesis-generating rather than causal. They suggest that hypothermia may warrant further investigation as a potential modifier of traditional risk factors, but definitive conclusions cannot be drawn from this exploratory analysis.

## Limitations

Treatment allocation was not randomized. Although statistical weighting reduced baseline imbalances, residual confounding from unmeasured variables may remain. The multicenter design could have introduced variability in case ascertainment, clinical decision-making, and supportive care. In addition, inverse probability of treatment weighting (IPTW) has inherent limitations. Extreme weights may arise when treatment paths are rare, increasing statistical noise and potentially destabilizing estimates. The validity of IPTW also depends on correct specification of the propensity score model; if relevant confounders are omitted or modeled incorrectly, bias may persist. Finally, IPTW cannot account for unmeasured confounding, and the effective sample size is reduced after weighting, which may limit precision.

The use of the Vermont Oxford Network database imposed inherent limitations on the granularity of specific clinical and surgical variables and on the precise temporal mapping of secondary outcomes, including intraventricular hemorrhage.

However, the absence of certain data, including feeding types, postnatal age at onset, and demographic characteristics, does not represent a significant limitation to the study’s objectives. Distinguishing between factors that influence the incidence of NEC and those that contribute to its progression from moderate to surgical disease is essential. Previous research indicates that progression to surgical intervention is primarily determined by the intensity of the localized inflammatory response and the extent of intestinal injury, rather than by pre-diagnostic dietary or baseline variables. Although the progression to surgical intervention in moderate NEC is multifactorial, more consistent predictors, such as prematurity and low birth weight, were included in the study analysis [39, 40].

Neurologic outcomes were assessed exclusively during hospitalization, which limits comparability because long-term neurodevelopmental evaluation was not performed. Similarly, long-term gastrointestinal outcomes such as intestinal failure, prolonged parenteral nutrition, and post-discharge growth were not evaluated. Future research with this cohort is planned to address these limitations, and results will be reported when available.

Data on early-onset sepsis from two control centers were excluded from this analysis due to differences in sepsis diagnostic criteria compared with those at the other centers. This decision was based on findings from the systematic data quality audit conducted before outcome analyses. The data from the remaining centers were homogeneous and balanced. Nevertheless, variations in data quality across centers may still introduce information bias in multicenter observational studies.

## Future directions

Long-term neurodevelopmental follow-up has been conducted to assess whether early clinical effects correspond to sustained improvements in neurological outcomes and growth. Analyses of inflammatory markers, thermography, proteomics, and lipidomics are ongoing within the NEOTHERM project.

The successful execution of a large randomized controlled trial requires the enrollment of several hundred infants per study arm, the establishment of multicenter collaboration, the implementation of standardized thermal management protocols, the employment of dedicated research nursing staff, and the assurance of comprehensive neuroimaging data collection. Efforts are underway to ensure this randomized project is feasible.

The application of a low-technology, servo-controlled cooling method, previously validated for safety [11, 12], enhances the generalizability of these findings to resource-limited and middle-income level III neonatal intensive care units (NICUs) managing very low birth weight (VLBW) infants with moderate NEC. Nevertheless, caution is warranted when extrapolating to contexts with differing staffing levels, monitoring capabilities, or surgical thresholds. Multicenter randomized trials in diverse settings are necessary to confirm external validity.

## Conclusions and relevance

This prospective cohort study demonstrated that adjuvant mild controlled hypothermia in preterm infants with moderate NEC was associated with lower rates of surgical intervention and reduced all-hospital mortality. These results suggest a potentially effective therapeutic approach for a severe condition that has lacked successful interventions to prevent disease progression for decades. The findings underscore the need for large-scale randomized clinical trials to confirm efficacy, evaluate long-term safety, and develop standardized treatment protocols.

## Supplementary information

Below is the link to the electronic supplementary material.

ESM 1 (DOCX 154 KB)

## Data Availability

Data will be made available upon reasonable request. All deidentified participant data will be provided to researchers who submit a methodologically sound proposal. Data access will be granted from 6 to 36 months following publication. Anonymized transcripts may be shared with researchers who meet the criteria for access to confidential data, pending approval by the Institutional Review Board (IRB).

## Abbreviations

aHR: Adjusted hazard ratio
aRR: Adjusted risk ratio
CI: Confidence interval
DHA: Docosahexaenoic acid
ELBW: Extremely low birth weight
GSH: Glutathione
HIE: Hypoxic-ischemic encephalopathy
IPTW: Inverse probability of treatment weighting
IVH: Intraventricular hemorrhage
LOS: Length of hospital stay
MNAR: Missing not at random
NEC: Necrotizing enterocolitis
NICU: Neonatal intensive care unit
NIPS: Neonatal Infant Pain Scale
PVL: Periventricular leukomalacia
SD: Standard deviation
SGA: Small for gestational age
SHR: Subdistribution hazard ratio
SIP: Spontaneous intestinal perforation
SIRS: Systemic inflammatory response syndrome
SMD: Standardized mean difference
SNAPPE-II: Score for Neonatal Acute Physiology with Perinatal Extension II
STROBE: Strengthening the Reporting of Observational Studies in Epidemiology
TLR4: Toll-like receptor 4
VLBW: Very low birth weight
VON: Vermont Oxford Network
