# Supplementary material for: Adjunctive mild controlled hypothermia for moderate necrotizing enterocolitis in very low birth weight infants: a prospective cohort study with external contemporaneous controls
Source: Eur J Pediatr. 2026 Oct 3;185(11):799. doi: 10.1007/s00431-026-07459-4 (PMC13634000; doi:10.1007/s00431-026-07459-4)
Supplement: Supplementary file 1 [file 431_2026_7459_MOESM1_ESM.docx]

**Figure SM-1: Database of control and intervention group within Vermont-Oxford Network**

**Table SM-1: Baseline Demographic and Clinical Characteristics of Study Participants by center (C).**

| **Charact** | **C.1** | **C.2** | **C.3** | **C.4** | **C.5** | **C.6** | **C.7** | **C.8** |
| --- | --- | --- | --- | --- | --- | --- | --- | --- |
| **Categorical Variables** [**No. (%)]** | | | | | | | | |
| Male Sex | 43/92 (46.7%) | 9/16  (56.2%) | 10/21  (47.6%) | 8/13  (61.5%) | 23/47  (48.9%) | 25/44  (56.8%) | 24/43  (55.8%) | 0/9  (0.0%) |
| Cesarean | 63/92  (68.4%) | 7/16  (43.7%) | 17/21  (80.9%) | 8/13  (61.5%) | 33/47  (70.2%) | 32/44  (72.7%) | 32/43  (74.4%) | 8/9  (88.8%) |
| SGA | 29/89 (33.6%) | 4/14  (28.5%) | 12/21  (57.1%) | 4/13  (30.7%) | 19/47  (40.4%) | 14/42  (33.3%) | 13/41  (31.7%) | 1/9  (11.1%) |
| Maternal Hypertension | 42/92 (45.6%) | 5/16  (25.0%) | 9/20  (45.0%) | 4/13  (30.7%) | 23/47  (48.9%) | 21/44  (48.8%) | 21/43  (48.8%) | 1/9  (11.1%) |
| Chorioamnionitis | 16/91 (17.5%) | 1/16  (6.2%) | 1/21  (4.7%) | 0/13  (0.0%) | 4/47  (17.5%) | 10/44  (8.5%) | 10/43  (23.2%) | 1/9  (11.1%) |
| Antenatal Steroid Use | 79/91 (86.8%) | 9/16  (56.2%) | 17/18  (86.8%) | 11/13  (94.4%) | 44/47  (93.6%) | 32/44  (72.7%) | 31/43  (72.0%) | 6/9  (66.6%) |
| Early-onset Sepsis ^e^ | 8/92 (8.7%) | 2/16  (12.5%) | 0/21  (0.0%) | 1/13  (7.6%) | 2/46  (4.3%) | - | 0/43  (0.0%) | - |
| **Continuous Variables [Mean (SD); Median (Q1, Q3)]** n=92/n=193 | | | | | | | | |
| Gestational Age (weeks) | 28.4 (2.6);  28.8 (26.4, 30.0) | 27.3  (3.31);  28.1 (25.7, 28.5) | 29.7  (2.7);  29.5 (27.8, 32.0) | 28.9 (3.5);  28.1 (26.4, 30.7) | 28.9 (2.4);  29.1 (26.8, 31.0) | 28.8 (2.7);  29.0 (27.0, 30.2) | 28.7 (2.8)  29.0 (27.0, 30.1) | 28.4 (4.0)  28.1 (25.7, 28.5) |
| Birth Weight (grams) | 957.9 (269.3);  922.5 (752.5, 1195.0) | 890.6 (268.3);  772.5 (690.0, 1085.0) | 1001.4 (258.0);  1030.0 (800.0, 1175.0) | 1031.4 (295.9);  970.0 (845.0, 1205.0) | 991.6 (236.5);  950.0 (784.0, 1200.0) | 1053.0 (248.9);  1032.5 (890.0, 1260.0) | 1055.0 (251.8);  1035.0 (875.0, 1275.0) | 962.1 (219.4);  986.0 (856.0, 1110.0) |
| SNAPPEII * | 29.8 (17.9)  29.0 (17.0, 41.0) | 20.0 (16.6)  17.0 (5.0, 32.5) | 38.5 (25.1)  33.0 (24.0, 50.0) | 33.8 (21.8)  30.0 (15.0, 44.0) | 19.2 (14.1)  15.0 (10.0, 29.0) | 28.4 (19.5)  30.0 (10.0, 42.0) | -- | 33.8 (33.2)  29.0 (17.0, 31.0) |
| Five-Minutes Apgar Score | 7.9 (1.8);  8.0 (7.0, 9.0) | 7.6 (1.0);  8.0 (7.0, 8.5) | 8.1 (1.2);  8.0 (8.0, 9.0) | 7.9 (1.5);  9.0 (7.0, 9.0) | 7.9 (1.5);  8.0 (7.0, 9.0) | 7.4 (1.8);  8.0 (7.0, 9.0) | 7.4 (1.8);  8.0 (7.0, 9.0) | 8.7 (1.2);  9.0 (9.0, 9.0) |

Notes: SD: Standard Deviation; Q1: First Quartile (25th percentile); Q3: Third Quartile (75th percentile).

a: P-values for categorical variables were calculated using Fisher's Exact Test.

b: P-values for continuous variables were calculated using the Wilcoxon Rank-Sum Test.

c: P-values for the comparison of baseline characteristics **across control centers** (C.2–C.8): male sex, p=0.92; cesarean, p<0.01; SGA, p=0.38; maternal hypertension, p=0.31; chorioamnionitis, p=0.07; antenatal steroid use, p<0.01; early-onset sepsis, p=0.15; gestational age, p=0.25; birth weight, p=0.23; SNAPPE-II, p<0.01; five-minute Apgar score, p=0.19.

d: P-values for between-center comparisons were calculated using the chi-square test for categorical variables and the Kruskal-Wallis test for continuous variables.

*: Center C7 does not utilize SNAPPE-II in clinical practice; consequently, data from this center are unavailable (43 missing data points).

e: Centers C6 and C8 were excluded from the early-onset sepsis analysis because the sepsis diagnostic criteria applied at these centers differed from those used by the remaining centers, precluding valid comparisons. This exclusion was decided during a systematic data quality audit conducted prior to the outcome analyses and independently of the study results. Data for these centers are shown for descriptive completeness only and were not included in the early-onset sepsis assessment. All other variables were harmonized across centers and included in the respective analyses. A center would only have been excluded from the study if the variables related to the study outcomes (death, surgery, cerebral hemorrhage, and leukomalacia) had differed from the predefined diagnostic classification; this did not occur.

**Figure SM-2: A Love Plot-Driven Analysis of Covariate Balance Improvement Following Inverse Probability of Treatment Weighting (IPTW) in Necrotizing Enterocolitis Surgery.**

**Table SM2.** **Neurological Outcomes in the Intervention and Control Groups: Sensitivity analysis.**

| **Outcome** | **Group** | | **Crude Risk Ratio (95% CI)** | **Adjusted Risk Ratio (95% CI)** |
| --- | --- | --- | --- | --- |
|  | **Hypothermia**  **n (%)** | **Control**  **n (%)** |  |  |
| Severe Intraventricular Hemorrhage (IVH)a | 10/92 (10.87%) | 9/162 (5.56%) | 1.95 (0.79; 4.93) | 2.16 (0.88; 5.27) |
| Severe Intraventricular Hemorrhage (IVH)-M1 | 10/92 (10.87%) | 24/193 (12.44%) | 0.87 (0.40; 1.78) | 1.03 (0.46; 2.29) |
| Severe Intraventricular Hemorrhage (IVH)-M2 | 10/92 (10.87%) | 40/193 (20.73%) | 0.52 (0.25; 1.01) | 0.69 (0.33; 1.43) |
| Severe Intraventricular Hemorrhage (IVH)-M3 | 10/92 (10.87%) | 9/193 (4.66%) | 2.33 (0.93; 5.87) | 1.70 (0.66; 4.38) |
| Periventricular Leukomalacia (PVL)b | 14/92 (15.22%) | 16/160 (10.00%) | 1.52 (0.73; 3.12) | 1.72 (0.86; 3.43) |
| Periventricular Leukomalacia (PVL)-M1 | 14/92 (15.22%) | 33/193 (17.10%) | 0.88 (0.46; 1.63) | 1.05 (0.55; 2.01) |
| Periventricular Leukomalacia (PVL)-M2 | 14/92 (15.22%) | 49/193 (25.39%) | 0.60 (0.32; 1.06) | 0.80 (0.43; 1.49) |
| Periventricular Leukomalacia (PVL)-M3 | 14/92 (15.22%) | 16/193 (8.29%) | 1.83 (0.88; 3.77) | 1.84 (0.86; 3.89) |

**Notes:** CI: Confidence Interval; a- Severe Intraventricular Hemorrhage ( ≥ grade III); b- Periventricular Leukomalacia ( grade III). M1: Controls who died prior to neuroimaging (N = 15 for IVH; N = 16 for PVL) were imputed as having the outcome (1), assuming early mortality was driven by severe neurological injury. Controls discharged without imaging (N = 16 for IVH; N = 17 for PVL) were imputed as outcome-free (0), assuming lack of imaging reflected clinical stability. M2: All missing controls (N = 31 for IVH; N = 33 for PVL) were imputed as having the outcome ($1$). M3: All missing controls (N = 31 for IVH; N = 33 for PVL) were imputed as having the outcome (0).
